# Foot Disorders, Foot Posture, and Foot Function: The Framingham Foot Study

**DOI:** 10.1371/journal.pone.0074364

**Published:** 2013-09-05

**Authors:** Thomas J. Hagedorn, Alyssa B. Dufour, Jody L. Riskowski, Howard J. Hillstrom, Hylton B. Menz, Virginia A. Casey, Marian T. Hannan

**Affiliations:** 1 Institute of Aging Research, Hebrew SeniorLife, Boston, Massachusetts, United States of America; 2 Department of Medicine, Harvard Medical School, Boston, Massachusetts, United States of America; 3 Institute of Applied Health Research, Glasgow Caledonian University, Glasgow, United Kingdom; 4 Leon Root MD, Motion Analysis Laboratory, Hospital for Special Surgery, New York, New York, United States of America; 5 Lower Extremity and Gait Studies Program, Faculty of Health Sciences, La Trobe University, Bundoora, Australia; University of South Australia, Australia

## Abstract

**Introduction:**

Foot disorders are common among older adults and may lead to outcomes such as falls and functional limitation. However, the associations of foot posture and foot function to specific foot disorders at the population level remain poorly understood. The purpose of this study was to assess the relation between specific foot disorders, foot posture, and foot function.

**Methods:**

Participants were from the population-based Framingham Foot Study. Quintiles of the modified arch index and center of pressure excursion index from plantar pressure scans were used to create foot posture and function subgroups. Adjusted odds ratios of having each specific disorder were calculated for foot posture and function subgroups relative to a referent 3 quintiles.

**Results:**

Pes planus foot posture was associated with increased odds of hammer toes and overlapping toes. Cavus foot posture was not associated with the foot disorders evaluated. Odds of having hallux valgus and overlapping toes were significantly increased in those with pronated foot function, while odds of hallux valgus and hallux rigidus were significantly decreased in those with supinated function.

**Conclusions:**

Foot posture and foot function were associated with the presence of specific foot disorders.

## Introduction

Foot disorders are common among older adults, with reported prevalence of some disorders as high as 65% [1]. Foot disorders and their related symptoms are associated with poor health outcomes, such as falls [2,3] and functional limitations [4,5]. However, the biomechanics of many foot disorders remain poorly understood. Few studies have systematically explored the anatomical or biomechanical factors associated with specific foot disorders.

Foot posture and function are thought to be associated with disorders such as hallux valgus [6], hallux rigidus [7], Tailor’s bunion [8], and hammer toes [9,10]. However, many of these associations are based on clinical observations and have not been objectively validated. A limited body of work suggests a plausible link. Previous studies have noted altered regional loading [11], significant differences in foot kinematics [12], and pronated foot function [13] in participants with hallux valgus. Similarly, several lower limb overuse conditions have been associated with variations in foot posture [14].

These prior studies are typically limited by the assessment of a small number of foot disorders and have often focused on specific sub-samples that may not reflect population norms [15,16]. The purpose of this study is to assess the relation between foot disorders, and foot posture and function in a population-based sample of adults.

## Methods

### Ethics Statement

The Framingham Foot Study has been approved by the Hebrew SeniorLife and Boston University Medical Center Institutional Review Boards and participants provided written, informed consent prior to enrollment.

### Population

Data were obtained from the Framingham Foot Study, a population-based assessment of foot health in older adults residing in the area of Framingham, Massachusetts, USA [17]. The sample was comprised of three groups: the Framingham Original Cohort [18], the Framingham Offspring Cohort [19], and Framingham community members recruited through census based random-digit dialing of residents over 50 years of age.

Data were collected from 2002 to 2008. Participants with foot posture, foot function, foot examination and covariate data were included. Participants with amputated feet were excluded from this analysis.

### Foot Examination

A trained examiner performed a standardized physical examination of participants’ feet to determine the presence of specific foot disorders in a manner comparable to a clinical assessment. In 1998, the reliability of the foot exam was tested in elderly residents of a long-term care facility (mean age 89). Comparisons of examiners produced kappa values >0.85 (all p<0.01), and all domains tested had excellent interobserver and intraobserver reliability. Disorders were recorded as either present or absent.

A weight-bearing assessment of participants’ feet was used to determine the presence of hallux valgus, hammer, claw and overlapping toes, and Tailor’s bunions. Hallux valgus was defined as a 15° or greater abduction of the hallux from the first metatarsal determined by comparison to a laminated depiction of the angle. Hammer toes were considered present if there was a contraction of the proximal interphalangeal joint. Claw toes were defined as a contraction of both the proximal and distal interphalangeal joint. Overlapping toes were considered present in any instance where a toe overrode an adjacent one. Tailor’s bunion was recorded when an enlargement and lateral displacement of the fifth metatarsalphalangeal joint was observed.

While participants were seated, the examiner assessed participants’ feet for plantar fasciitis, Morton’s neuroma, and hallux rigidus. To determine presence of plantar fasciitis, the examiner applied four pounds of force to the insertion of the plantar fascia on the plantar surface the foot and recorded the disorder as present if the participant reported feeling pain. Hallux rigidus was considered present if the hallux was frozen or rigid during attempted passive movement by the examiner. Morton’s neuroma was assessed by exerting four pounds of force between toes two and three, three and four, and then squeezing all toes simultaneously. If the participant reported pain during any part of this procedure, Morton’s neuroma was recorded as present.

During the clinical examination, other variables of interest were obtained, including age, sex, height as measured by a calibrated stadiometer, and weight as measured by a calibrated balance beam scale.

### Plantar Pressure Measurement

Plantar pressure data were collected using a Tekscan Matscan (Tekscan Inc., Boston, MA) at 40 Hz. This pressure mapping system has demonstrated good measurement reliability [20]. Participants were instructed to walk along a path containing the mat at a self-selected pace. Scans were collected using the two-step method [21]. The two-step method involves participants stepping on the pressure mat with their second step and has been shown to be as reliable as the mid-gait approach [22]. There were two trials to collect one scan per foot while walking. A scan with participants standing in a self-selected bipedal, weight-bearing stance was also collected.

### Biomechanical Measures

Foot posture and function were assessed using participants’ plantar pressure scans. Foot posture was characterized using the modified arch index (MAI) [23], measured from participants’ standing scan. MAI is calculated by dividing the length of the foot, minus the toes, into three equal portions, and dividing the pressure in the middle third by that of all three regions. MAI is correlated with other measures of foot posture, notably navicular height [24].

Foot function was characterized using the center of pressure excursion index (CPEI), a measure of foot function throughout the gait cycle. CPEI is defined as the distance between a construction line drawn from the first and last points of each foot’s center of pressure trajectory and the center of pressure at the distal third tertile of the foot. This value is normalized by foot width and multiplied by 100 to obtain a percentage excursion of the center of pressure. CPEI has been shown to be sensitive to changes in static foot alignment [25].

**Table 1:** Participant characteristics and prevalence data of the specific foot disorders including in the Framingham Foot Study, 2002-08.

**Characteristic**	**Mean (Std Dev**)
Age (years)	66.2 (10.5)
Body Mass Index (kg/m^2^)	28.4 (5.5)
Weight (lbs)	174.1 (39.4)
Height (in)	65.5 (3.9)
**Foot Disorder**	**Prevalence N (%**)
Hallux Valgus	1472 (26.3)
Hammer Toes	894 (16.2)
Morton’s Neuroma	439 (8.0)
Overlapping Toes	294 (5.3)
Tailor’s Bunion	197 (3.6)
Plantar Fasciitis	177 (3.2)
Hallux Rigidus	173 (3.1)
Claw Toes	74 (1.3)

### Statistical Methods

The distribution of CPEI and MAI were divided in quintiles. For CPEI, feet in the top and bottom quintile were defined as having supinated and pronated foot function, respectively. For MAI, the top and bottom 20% were considered pes planus and pes cavus feet, respectively. The middle 60% of each measurement was used as the referent group in our analyses.

A per-foot analysis was used to analyze the relation of foot posture and function to the foot disorders included in our study. Logistic regression using Generalized Estimating Equations (GEE) to control for the correlation between participants’ two feet was used to determine odds ratios and 95% confidence intervals for the relation between specific foot disorders, foot posture, and foot function. Both crude and adjusted (age, sex, height, and weight) models were computed.

## Results

**Figure 1:**
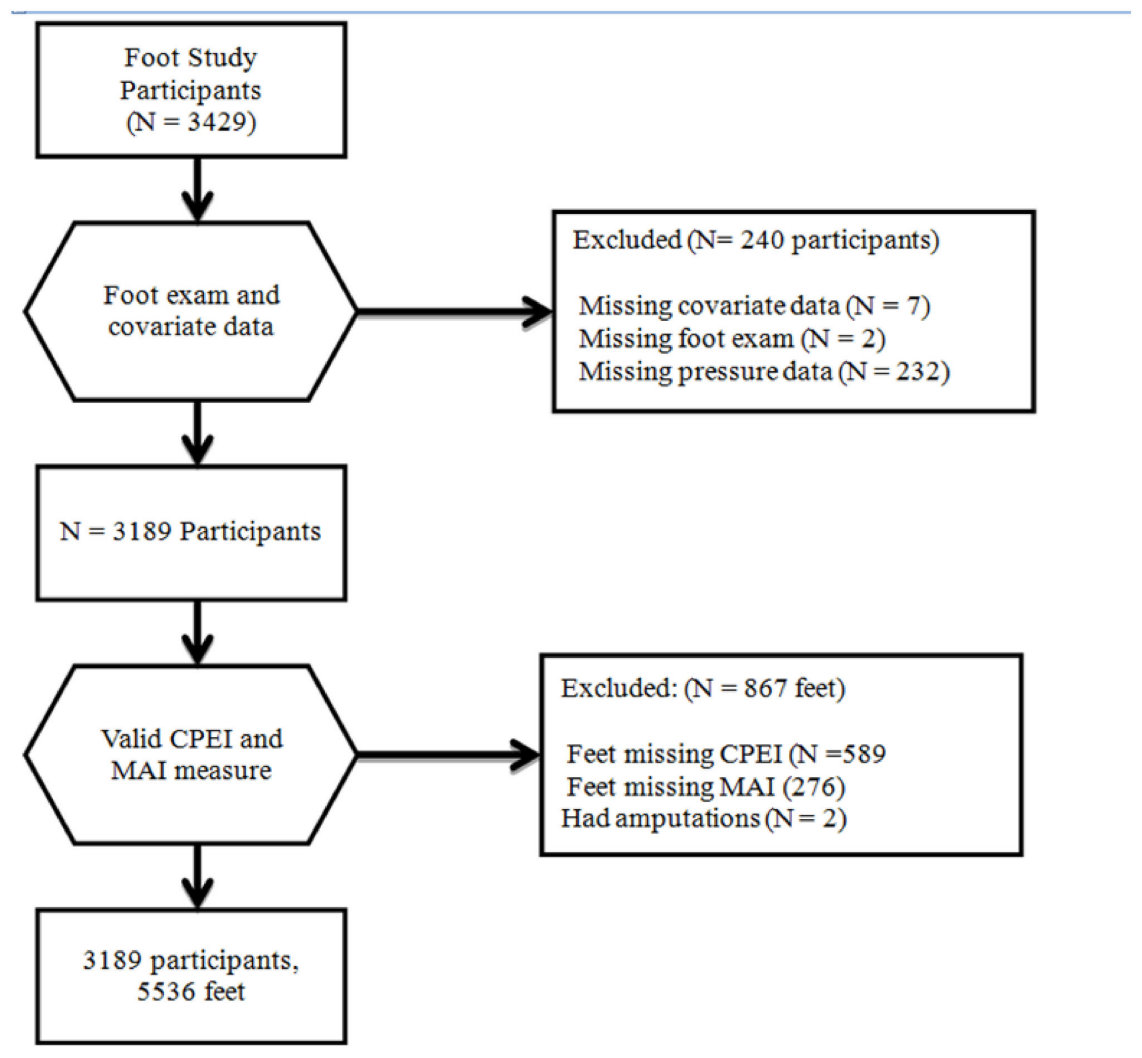
Inclusion flow chart.

Of 3,429 participants in the Framingham Foot Study, 3,189 participants completed foot examinations and had valid pressure scans contributing 5,536 feet to this analysis (Figure 1). The participants had a mean age of 66.2 years and mean BMI of 28.4 kg/m^2^, with 56% of the sample was female (Table 1). The most prevalent foot disorders were hallux valgus (26.3% of feet) and hammer toes (16.2% of feet), while the least prevalent disorder was claw toes (1.3% of feet).

Foot posture, as measured by modified arch index (MAI), was significantly associated with specific foot disorders (Table 2). Unadjusted models show a significant, positive association between hammer toes and feet in the planus group (p = 0.0007), a relation preserved in adjusted models (p = 0.0003). Although the unadjusted model was not significant (p = 0.072), in the adjusted model, planus feet showed a significant positive relation with overlapping toes (p = 0.018). There were no significant relations observed between cavus feet and the foot disorders that were assessed in our study.

**Table 2:** Unadjusted and adjusted* odds ratios and 95% confidence intervals (CI) for relation of foot disorders to foot posture as defined by modified arch index (MAI).

	**Unadjusted Odds Ratios (95% CI)**		**Adjusted Odds Ratios (95% CI)**	
	**Planus**	**Cavus**	**Planus**	**Cavus**
Hallux Valgus	0.95 (0.83, 1.10)	0.96 (0.84, 1.10)	1.05 (0.90, 1.22)	0.88 (0.77, 1.02)
Hammer Toes	**1.32 (1.13, 1.55)**	0.99 (0.82, 1.19)	**1.38 (1.16, 1.65)**	.94 (0.78, 1.13)
Morton’s Neuroma	1.27 (0.99, 1.62)	1.15 (0.91, 1.47)	1.11 (0.86, 1.44)	1.25 (0.97, 1.61)
Overlapping Toes	1.30 (0.98, 1.72)	1.05 (0.79, 1.40)	**1.44 (1.06, 1.94)**	0.95 (0.71, 1.27)
Tailor’s Bunion	1.19 (0.85, 1.68)	1.17 (0.88, 1.55)	1.27 (0.88, 1.84)	1.10 (0.83, 1.46)
Plantar Fasciitis	1.14 (0.78, 1.66)	1.08 (0.73, 1.58)	0.96 (0.66, 1.42)	1.22 (0.82, 1.81)
Hallux Rigidus	1.24 (0.93, 1.64)	0.85 (0.58, 1.24)	1.26 (0.92, 1.73)	0.84 (0.57, 1.23)
Claw Toes	0.79 (0.44, 1.41)	0.85 (0.46, 1.57)	0.79 (0.43, 1.44)	0.81 (0.44, 1.51)

* multivariate GEE models adjusted for effects of age, sex, height, and weight

Foot function, measured by center of pressure excursion index (CPEI), was associated with specific disorders (Table 3). In unadjusted models, pronated feet were positively associated with hallux valgus, while a negative association was noted in those with supinated feet. However, in adjusted models, only with the relation between supinated foot function and hallux valgus was preserved (p = 0.0045). Overlapping toes had a significant positive relation with pronated feet in both unadjusted (p = 0.0030) and adjusted (p = 0.0099) models, but there was no significant difference among supinated feet. Feet with supinated function were negatively associated with hallux rigidus (p = 0.003).

## Discussion

The purpose of this study was to evaluate the relation between foot disorders, posture, and function in a population-based cohort. Both planus foot posture and pronated foot function were associated with significantly higher odds of several foot disorders. Cavus foot posture, as defined by modified arch index (MAI), did not affect the risk of foot disorders in this population, while supinated foot function was associated with a reduced risk of both hallux valgus and hallux rigidus. These results support the widely-held clinical view that pronated foot function contributes to an increased risk of foot disorders in adults.

Clinical theory predicts an association between planus feet, pronated function, and hallux valgus [26]. However, studies have yielded inconsistent findings regarding the associations of hallux valgus with foot posture and function. In a systematic review and meta-analysis of papers analyzing foot posture and hallux valgus, Nix et al. found no consistent association between hallux valgus and clinical measures of foot posture, such as the arch index [27]. We also found that hallux valgus was not significantly associated with foot posture as measured by the MAI. Previous studies of dynamic foot function in hallux valgus have also yielded conflicting results. Research has reported that hallux valgus is [28] and is not [29] associated with pronated foot function. In our current study, unadjusted models showed significantly higher odds of having hallux valgus among feet with pronated function, but this result was attenuated after adjustment for covariates. Supinated function was associated with significantly reduced odds of having hallux valgus. The finding that pronated feet did not have higher odds of hallux valgus conflicts with previous research [28]. One reason for the discrepancy may be related to the measure of foot function. In the Kernozek et al. study, the researchers used hindfoot alignment to quantify foot function, whereas our study utilized center of pressure excursion index (CPEI) for function. CPEI is a dynamic measure of foot function, and while it has been shown to relate to static evaluation of foot posture [30], these results obtained from static measures may not be directly comparable to dynamic measures of foot function. Moreover, as no measure of hallux valgus severity was collected, it is possible that the more mild cases of hallux valgus (e.g., 15-25° of abduction) obscure relations between severe cases (greater than 25° of abduction [31]) and pronated foot function.

Clinical texts note planus foot posture and pronated function as possible causes of hallux rigidus [7]. However, Zammit et al. reported no strong association between hallux rigidus and measures of foot posture in a systematic review of case control studies [32]. We also found that foot posture measured by MAI was not significantly associated with hallux rigidus. Previous studies [33] [34] have noted increased loading of the hallux, lesser toes [33,34] and lateral metatarsals [34] in those with hallux rigidus. In our study, individuals who walked with supinated foot function had significantly reduced odds of hallux rigidus in adjusted models, which conflicts with the previous finding of greater lateral loading [34]. However, the Bryant et al. [34] examined a smaller sample of participants and used pressure measures that may not directly indicate pronated or supinated foot function. Given that individuals with supinated feet had significantly lower odds of having hallux valgus and hallux rigidus, these results may suggest that these disorders are more common in feet that are relatively pronated.

**Table 3:** Unadjusted and adjusted* odds ratios and 95% confidence intervals (CI) for relation of specific foot disorders and foot function defined by center of pressure excursion index (CPEI).

	**Unadjusted Odds Ratios (95% CI)**		**Adjusted Odds Ratios (95% CI)**	
	**Pronator**	**Supinator**	**Pronator**	**Supinator**
Hallux Valgus	**1.15 (1.01, 1.30)**	**0.78 (0.69, 0.89)**	1.08 (0.95, 1.22)	**0.82 (0.71, 0.94)**
Hammer Toes	0.92 (0.79, 1.06)	0.93 (0.79, 1.09)	0.91 (0.78, 1.05)	0.97 (0.82, 1.15)
Morton’s Neuroma	0.85 (0.67, 1.08)	0.96 (0.76, 1.22)	0.84 (0.66, 1.06)	1.02 (0.80, 1.29)
Overlapping Toes	**1.47 (1.14, 1.89)**	1.00 (0.75, 1.33)	**1.40 (1.08, 1.80)**	1.06 (0.79, 1.42)
Tailor’s Bunion	1.07 (0.79, 1.46)	0.80 (0.60, 1.07)	1.03 (0.76, 1.39)	0.83 (0.62, 1.13)
Plantar Fasciitis	1.00 (0.70, 1.43)	1.21 (0.89, 1.66)	1.02 (0.71, 1.47)	1.24 (0.91, 1.71)
Hallux Rigidus	1.16 (0.92, 1.48)	**0.58 (0.41, 0.83)**	1.20 (0.93, 1.54)	**0.57 (0.39, 0.83)**
Claw Toes	1.12 (0.71, 1.75)	0.83 (0.48, 1.43)	1.12 (0.71, 1.76)	0.87 (0.49, 1.54)
* multivariate GEE models adjusted for effects of age, sex, height, and weight				

The biomechanics of lesser toe deformities have not been extensively investigated. Clinical texts note that the presence of hallux valgus [26], pes planus [26] and pes cavus [10] are potential risk factors for hammer toes due to the formation of muscle imbalances in the foot [9,26,35]. In our study, planus foot posture was associated with increased odds of hammer toes in both crude and adjusted models. One reason for this limited agreement with clinical risk factors is that different pathways may be responsible for the development of hammer toes in planus and cavus feet [35]. As this study included mostly older adults, who typically have lower arches than younger adults [36,37], it is likely that hammer toes related toplanus foot posture are prevalent in this population. Our results suggest that foot posture rather than foot function is associated with hammer toes. Longitudinal and prospective studies are needed to fully understand the risk factors of hammer toes.

After adjustment for covariates in our study, both planus foot posture and pronated function were associated with increased odds of overlapping toes. Overlapping toes are a well recognized consequence of severe hallux valgus [26], despite the lack of objective research into the relations between overlapping toes and foot biomechanics as well as a lack of understanding with regard to the natural history of hallux valgus. Though this study did not examine if foot disorders were concomitant, it was noted that pronated function was associated with both overlapping toes and hallux valgus in unadjusted models. If the presupposed hypothesis that pronated feet have more severe hallux valgus relative to the referent, sufficient severity of hallux valgus may be a risk factor of overlapping toes.

While clinical work suggests that plantar fasciitis is associated with both a cavus [16] and planus [38] foot posture, Irving et al found no conclusive link between chronic heel pain, foot posture or function across seven studies reviewed [39]. In the current study there was no significant relation between plantar fasciitis, foot posture, and foot function. Plantar fasciitis was rare in this population (3.2%), but other factors may have contributed to this null result. As factors such as time spent standing [40] are often cited as potential causes of plantar fasciitis, this population may not have had the appropriate exposures. Moreover, as individuals between 40 and 60 years of age are thought to have the highest risk [38] a significant portion of our population can be classified as low risk.

A limited body of evidence suggests that cavus foot posture and supinated function play a role in the formation of claw toes [26], while pronated foot function is a risk factor for Tailor’s bunions [8] and Morton’s neuroma [26,41]. In this study, Tailor’s bunions, Morton’s neuroma, and claw toes were not associated with either foot posture or function in adjusted models. The null result associated with these disorders is likely due to their low prevalence in our population.

### Strengths and Limitations

Although we noted significant associations of foot posture and function with specific foot disorders, there are several strengths and limitations that must be considered. First, the data were cross-sectional; meaning no causal relation between foot posture, function, and disorders can be inferred. Longitudinal research is needed to better understand the causality between foot posture and function to foot disorders. Another limitation is possible measurement error due to the collection of only a single plantar scan for each foot during walking and in quiet stance. However, this limitation is greatly mitigated by the large number of participants in the study population. Additionally, although the measures of foot posture and function used in this study are not clinically common, CPEI [30] and MAI [23] are related to clinical measures. Direct measurement of the foot posture through radiographic evidence or foot function through a kinematic assessment may provide further information regarding foot posture and function.

There were also several important strengths to the study. To our knowledge this is the first study to evaluate the relation between foot posture, function, and common foot disorders at the population level, offering a broad picture of foot disorders as they occur in community-dwelling adults. This study also included a large population of older adults, a group that is greatly affected by foot disorders, but whose biomechanics are not as commonly studied in foot research.

## Conclusions

Foot posture and dynamic foot function were both associated with specific foot disorders in this cross-sectional study population. Our results are generally in agreement with biomechanical theory and clinical observations of specific foot disorders. Planus foot posture and pronated foot function were both positively associated with several foot disorders, supinated foot function was negatively associated with several foot disorders. These results underscore the utility of clinical input in understanding the relations between foot posture, function, and foot disorders. Future work should investigate the factors contributing to the onset of foot disorders, and evaluate interventions to offset biomechanical differences that coincide with their presence.
